# A Computational Approach to Analyzing Spatiotemporal Trends in Gun Violence and Mental Health Disparities among Racialized Communities in US Metropolitan Areas

**DOI:** 10.1007/s11524-025-00976-x

**Published:** 2025-04-22

**Authors:** Fahimeh Mohebbi, Amir Masoud Forati, John R. Mantsch, Madeline Campbell, Rina Ghose

**Affiliations:** 1 College of Engineering and Applied Science, University of Wisconsin-Milwaukee, Milwaukee, WI USA; 2 Department of Medicine, University of Wisconsin-Madison, Madison, WI USA; 3 Department of Pharmacology & Toxicology, Medical College of Wisconsin, Milwaukee, WI USA; 4 Center for Sustainability, Health, and the Environment, Medical College of Wisconsin, Milwaukee, WI USA

**Keywords:** Mental health inequities, Gun violence, Entropy statistics, Time series analysis

## Abstract

Gun violence is a leading cause of death and injuries in the USA, adversely affecting physical and mental health among its survivors. Declared as a public health crisis in 2024, It disproportionately affects African Americans. It is linked to discriminatory policies like “redlining,” which fostered racial segregation and systemic inequities, perpetuating cycles of violence and mental health disparities. This study explores the relationships between racial segregation, systemic inequities, gun violence, and mental health through a data-driven, longitudinal study (2005–2021) of Milwaukee, WI, a hyper segregated metropolitan region. Our investigation aims to inform evidence-based, place-sensitive policies to promote social justice, reduce disparities, and foster healthy communities. Utilizing location-based demographic and socio-economic data from the U.S. Census, gun violence data from the Wisconsin Incident-Based Reporting System, and mental health data from the CDC’s PLACES dataset, we conduct spatial and temporal analyses and geovisualization in GIS. To understand trends and correlations, we conduct time series decomposition, Mann–Kendall trend tests, and entropy statistics. Our findings indicate that racially segregated neighborhoods experience higher rates of gun violence and poorer mental health outcomes. Predominantly African American neighborhoods exhibit patterns of “consecutive,” “sporadic,” and “new” hotspots of gun violence, while predominantly white neighborhoods are characterized as “cold spots.” Physical and mental health disparities in Milwaukee indicate similar patterns. The results of this study highlight the profound impact of historical and systemic socioeconomic discrimination on contemporary public health issues.

## Introduction

Gun violence is a leading cause of death and injuries in the USA, adversely affecting the survivors’ physical and mental well-being. With approximately 13,000 deaths and 75,000 non-fatal injuries annually [1], gun violence represents a significant public health crisis. Gun violence is highly racialized,it disproportionately affects Black men, who are 14 times more likely to die from gun violence than white men (CDC). Firearm homicide is the leading cause of death among Black males aged 15–35 [1]. Similarly, Black children face higher exposure to gun violence as compared to other races [2]. This racialization of gun violence is closely linked to systemic poverty, inequality, and racial segregation [3], [4].

Racial segregation in urban centers is an outcome of “redlining,” a discriminatory mortgage lending policy that denied minority populations equal access to loans and housing [5]. Neighborhoods inhabited by African Americans and other minorities or immigrants were often graded lowest on Homeowner's Loan Corporation (HOLC) maps from the 1930s [6]. Despite the 1968 Fair Housing Act, weak enforcement allowed continued mortgage discrimination, confining African Americans into low-income neighborhoods, thus exacerbating racial economic disparities [7]. Deindustrialization and disinvestment processes further contributed to concentrated poverty, inequality and high rates of crime and gun violence within the segregated neighborhoods, limiting access to essential resources and services, and perpetuating health disparities [8]. The significance of place-based factors upon health outcomes has been well recognized by health agencies, which have emphasized place-sensitive inquiries at the neighborhood scale. Pettygrove and Ghose [9] emphasized the importance of scale in urban food justice research, highlighting how localized, neighborhood-level analyses can reveal disparities that broader, city-wide studies may overlook. Their findings underscore that examining the food environment at the community level is critical because the quality and accessibility of food can vary significantly within a single neighborhood. This localized approach is essential for city planners and community organizers aiming to address the public health crisis, which is exacerbated by spatial inequalities [9].

The association between socioeconomic disparities, crime, neighborhood violence, and poor mental health is increasingly prioritized [4, 6, 10–13]. While living in high-crime neighborhoods increases the stress levels of residents, experiencing gun violence heightens the risk of mental health issues such as depression and anxiety [12], leading to developmental challenges among the youth [14]. Therefore, inquiries into neighborhood-level conditions regarding mental health are increasingly prioritized [15].

This study employs a socioecological model, which posits that individual health behaviors and outcomes are significantly impacted by a complex interplay of interpersonal, neighborhood, societal, and geographic factors. It explores the complex associations between gun violence, mental health, and historical redlining. Utilizing location-based demographic data from the U.S. Census, gun violence data from the Wisconsin Incident-Based Reporting System, and mental health data from the CDC’s PLACES dataset, the study conducts spatial and temporal analyses through GIS. The findings aim to inform evidence-based policies to promote social justice, reduce disparities, and foster healthier, more equitable communities.

The study site, Milwaukee County, Wisconsin, home to 918,661 residents, is a diverse region with 58.9% identifying as White, 26.3% as Black, and 15.6% as Hispanic or Latino (U.S. Census Bureau, 2020). The county includes Milwaukee City, known for its hyper-segregation and significant rise in gun violence [16, 17], which exceeds the national average in homicides, assaults, and shootings [18]. Contributing factors encompass socioeconomic inequalities and greater accessibility to firearms, both of which are associated with elevated violent crime rates in urban areas [16, 19–21]. This violence impacts community well-being and strains law enforcement resources [22]. Despite community initiatives and law enforcement efforts, the crisis persists, requiring a multifaceted approach involving policy reform, community engagement, and enhanced access to social services. Milwaukee’s history of racial segregation and income inequality makes it a compelling case study for exploring the intersectionality of gun violence, mental health, and racial disparities [8], [23].

## Methods

To understand the interactions between health and place and to formulate place-sensitive policies, the Centers for Disease Control (CDC) has emphasized a spatial approach. Therefore, we employ a place-sensitive and context-dependent multi-dimensional and multi-scalar spatial methodology drawn from our decade-long investigations into racialized poverty, food insecurity, physical and mental health disparities, and opioid overdose crisis. To conduct spatial statistics, modeling, and geovisualization, we used Geographic Information Systems (GIS), a specialized computer system for capturing, storing, analyzing, and displaying location-based big data. We collected geo-referenced demographic and socioeconomic data from the 2020 U.S. Census (via Data.census.gov) and organized it at the census tract level. Using census tracts as the analytical scale enables precise spatial resolution, allowing for detailed examination of neighborhood-level variables.

Our data on firearm-related incidents at the census tract level is derived from the Wisconsin Incident-Based Reporting System (WIBR) between 2005 and 2021 [24]. This data allowed us to categorize and analyze firearm involvement across various criminal activities (arson, assault, burglary, criminal damage, homicide, and vehicle theft). A lexicon-based classifier, a natural language processing (NLP) technique, was employed to identify offenses involving firearms, detecting entries where weapons like handguns, rifles, and other guns were used.

Data on physical and mental health outcomes were sourced from the CDC’s PLACES dataset (2014–2018). Poor physical health was defined as the percentage of adults reporting 14 or more days of poor physical health in the past month, and poor mental health was measured as the percentage of adults reporting 14 or more days of poor mental health in the same period. These metrics were calculated at the census tract level, providing localized insights into health disparities. The CDC generated these small-area estimates through a multilevel regression and poststratification approach, which enables precise tract-level data despite potential limitations in survey sample sizes [25] (CDC PLACES, 2025, [25].

All variables were collected, processed, and integrated into the Milwaukee County census tract shapefile from the TIGER/Line database (www.census.gov) in ArcGIS Desktop 10.7. This spatial framework allowed us to analyze data at multiple geographic scales—census tract, municipal, and county levels. Our study mapped the distribution of gun violence and mental health issues while highlighting the socioeconomic and racial factors shaping these urban phenomena.

Spatial statistics and geovisualization of social determinants of health were used to analyze patterns, clusters, and variations of geospatial determinants of health (GDOH) across multiple geographic scales. The results identify high-risk neighborhoods and assess their demographic, socio-economic, and health characteristics to inform place-sensitive interventions. This approach highlights areas with racialized health inequities and disparities, guiding data-driven policy interventions essential for health equity research and advocacy.

### Temporal Dynamics of Gun Violence

To examine the temporal dynamics of gun violence, we synthesized both yearly and monthly time series data, capturing the intricate patterns of gun violence incidents in Milwaukee. Utilizing the Python statsmodels package [26], we decomposed the overall aggregated time series to dissect the temporal dynamics of gun violence into their fundamental components: the overarching trend, the cyclic seasonality, and the irregular remainder (noise).

Further refining our focus to assess the impact of historical redlining, we segmented our analysis based on the designated redlining grades, creating four distinct time series, each corresponding to a specific redlining grade, for a granular examination of violence trends within these demarcated areas. We then decomposed each series, revealing their respective trends, seasonal variations, and residual elements.

To quantitatively compare the gun violence trends across different redlining grades and evaluate the significance of their disparities, we applied a statistical test for equality of regression coefficients [27], thereby assessing the rate of change in gun violence incidents over time for each redlining category. This statistical analysis aimed to ascertain whether the vestiges of redlining continue to exert an influence on the present-day patterns of gun violence, with significant results implying that the historical implications of redlining might still be influencing the contemporary landscape of gun violence.

### Mapping Gun Violence Trends

To visualize and analyze the spatiality of gun violence, incidents were mapped at the census tract level. The Mann–Kendall (MK) statistic was then employed to analyze the trend of gun violence incidents in each neighborhood [28]. MK statistic is a non-parametric test to detect consistent trends in time-series data. Here, it facilitated the identification of areas where gun violence was either escalating, diminishing, or remaining relatively static over the observed period.

Next, utilizing ArcGIS Pro, an emerging hotspot analysis was undertaken to understand the spatial distribution and evolution of gun violence incidents over the study period. The analysis evaluates trends in the context of both space and time, identifying areas with statistically significant increasing or decreasing trends in gun violence. The analysis involves breaking the data into spatial neighborhoods, which are then assessed across the temporal span. The results categorize neighborhoods based on the nature and persistence of their hotspots. The potential categories are listed below.

- New hot spot: An area that has recently become a hotspot, indicating an escalation in gun violence.
- Persistent hot spot: An area consistently identified as a hotspot over the study period, signifying a sustained high level of gun violence.
- Diminishing hot spot: A previously identified hotspot showing a decrease in gun violence trends.
- Oscillating hot spot: An area that has fluctuated between being a hotspot and a cold spot over the study period.
- New and historical cold spots: Areas exhibiting consistently low or declining levels of gun violence.

By categorizing areas in such a manner, the emerging hot spot analysis provides insights into the evolving landscape of gun violence, allowing for targeted interventions.

### Analyzing The Nexus Between Mental Health and Gun Violence

The relationship between gun violence and mental health is complex and cannot be fully captured by traditional regression techniques alone. Regression methods are typically used to estimate direct associations between variables, relying on predefined models that assume linearity or specific types of relationships. While valuable, these methods may overlook non-linear, non-monotonic, or interdependent dynamics that exist between variables. To address these limitations, this study employs joint entropy as a statistical tool to capture the complexity of the relationship between gun violence and mental health.

At its core, entropy is a statistical measure that quantifies the amount of uncertainty or randomness in a variable [29]. Joint entropy provides a measure of the combined uncertainty or dependence between two variables, in this case, gun violence (*X*) and mental health (*Y*). It calculates the degree to which changes in one variable correspond to changes in the other by incorporating their joint probability distribution. This approach allows for the identification of intricate patterns in the data, such as non-linear dependencies, that may not be apparent using regression alone [30]. For two variables *X* (gun violence) and *Y* (mental health), joint entropy $H\left(X,Y\right)$ is calculated as follows:

$$H\left(X,Y\right)=-\sum_{i=1}^{n}\sum_{j=1}^{m}P({x}_{i},{y}_{j})\text{log}P({x}_{i},{y}_{j})$$

where it represents the joint probability of observing specific values of gun violence and mental health. By analyzing this joint distribution, entropy offers insights into the underlying structure and variability of their relationship.

In this study, a higher joint entropy value signifies a more complex relationship between gun violence and mental health. This indicates that the variability in gun violence and mental health cannot be easily explained by simple linear relationships or deterministic patterns. Multiple interacting factors such as socioeconomic conditions, historical redlining practices, and community dynamics may contribute to this complexity. In such cases, the relationship between the two variables is less predictable, with variations in one not consistently corresponding to variations in the other. Conversely, a lower joint entropy value indicates a more deterministic or predictable relationship. This occurs when changes in one variable strongly align with changes in the other, suggesting a clearer and more direct association. In neighborhoods with consistent socioeconomic conditions, the relationship between gun violence and mental health may be more straightforward and easier to predict. When there is no meaningful relationship between gun violence and mental health, the joint entropy reflects independent randomness. In such cases, variations in gun violence provide no predictive information about variations in mental health and vice versa. This scenario results in an entropy value that primarily represents noise or lack of structure in the joint distribution of the variables. ArcGIS Pro Local Bivariate Relationships tools were utilized for analysis, which categorizes each feature into one of six relationship types: not significant, positive linear, negative linear, concave, convex, or undefined complex [29].

### Incorporation of Redlining Maps for Analysis

Redlining maps, created in the 1930s to assess investment risk, significantly shaped the racial demographics and socioeconomic conditions of urban neighborhoods [5], [6]. Based on demographics, neighborhoods were systematically graded from “A” (most desirable) to “D” (least desirable), profoundly influencing their investment patterns, mortgage lending, and infrastructure development. Neighborhoods classified as D and C became dominantly African American and Hispanic neighborhoods, while A and B neighborhoods became White enclaves. This discriminatory practice entrenched segregation, lowered property values, and restricted access to essential services in minority neighborhoods, perpetuating poverty and limiting opportunities across generations.

**Fig. 1:**
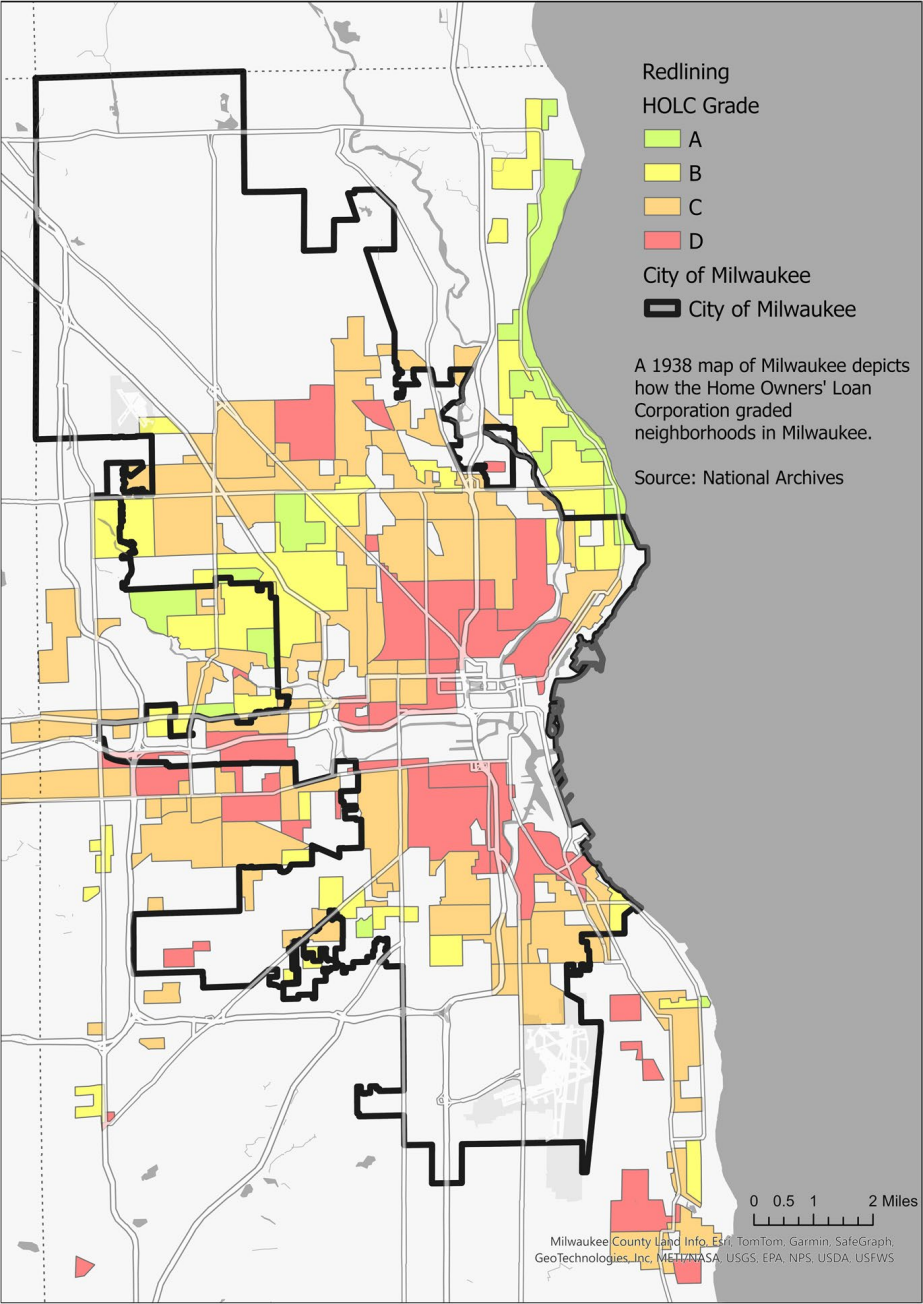
Redlining map of Milwaukee City

To integrate the historical context of redlining into our analysis, we categorized gun violence incidents based on historical redlining grades, creating four distinct time series corresponding to these grades. Time series decomposition was applied to break down the data into its fundamental components: long-term trends, seasonal variations, and random fluctuations [26]. This methodological approach provided insights into unique patterns of gun violence associated with each redlining grade. Figure 1 illustrates the spatial distribution of these grades and their lasting impact on urban development and socioeconomic disparities. By examining these time series, we aimed to uncover disparities in gun violence trends attributable to historical redlining practices.

## Results

Analyzing gun violence in Milwaukee on yearly and monthly scales reveals periodic fluctuations, indicating both seasonal trends and long-term shifts. Since 2013, incidents have increased, with a significant rise from 2018 to 2021, peaking at 10,793 in 2021. Monthly data show a consistent pattern of higher incidents during summer (June–August) compared to colder months, though variations exist over the years. The data highlights an intensification of gun violence in Milwaukee, particularly over the last 4 years.

**Fig. 2:**
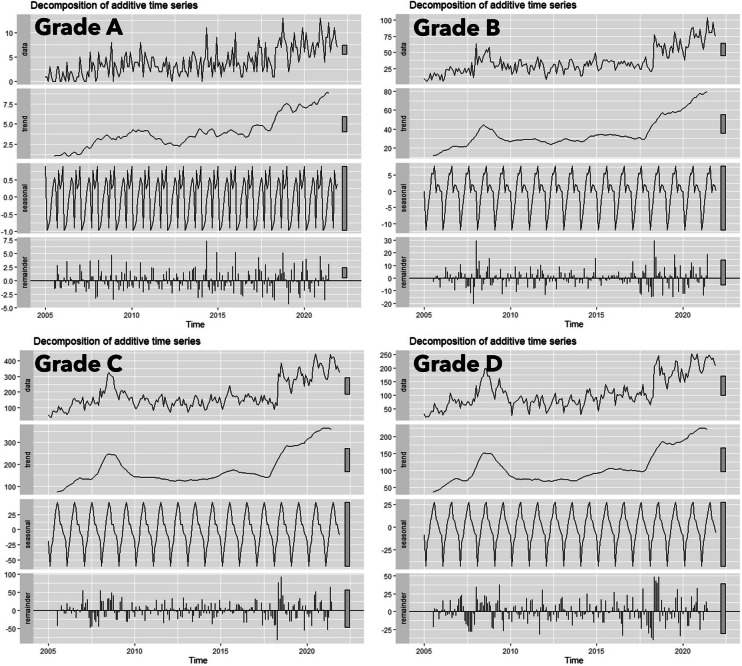
Time series decomposition of gun violence data (2005–2021) across Milwaukee neighborhoods stratified by historical redlining grades (**A**, **B**, **C**, and **D**). Each panel illustrates the extraction of three components: trend (long-term patterns), seasonality (recurring temporal cycles), and residual noise (stochastic variability). Using computational models, this analysis reveals significant disparities in temporal dynamics associated with historical redlining practices, emphasizing pronounced differences in gun violence trends across neighborhoods with varying grades

To examine gun violence in the context of neighborhood settings, we analyzed incidents categorized by historical redlining grades, creating four distinct time series across Milwaukee (Fig. 2). Each time series corresponds to one of the redlining grades (A, B, C, and D). Using a decomposition method, we further analyzed these time series by breaking them down into three fundamental components: trend, seasonality, and residual noise. The trend component highlights long-term patterns and underlying changes over time, while the seasonal component captures periodic fluctuations, providing insights into recurring patterns. The residual noise component isolates random variations, allowing us to assess the stability of the data. The results of the decomposition, illustrated in the accompanying figure, enable a comparative analysis of the temporal dynamics across neighborhoods with differing redlining grades.

Grade A neighborhoods, with a population of 11,734, experienced 840 gun incidents, resulting in a gun violence rate (GVR) of 7158.68 per 100,000. The trend slope, indicating the rate of change in incidents, was 841.64. In grade B, with a population of 55,768, incidents increased to 7436, yielding a GVR of 13,333.81 and a sharper trend slope of 1260.37, indicating a more rapid increase.

The most concerning findings are in grades C and D neighborhoods. Grade C, with a population of 190,827, experienced 37,463 incidents, leading to a GVR of 19,631.92 and a slope of 1523.86. Grade D, with a smaller population of 119,373, reported 22,687 incidents, resulting in a GVR of 19,005.14 and the highest slope of 1677.70 among all grades.

Table 1 demonstrates a progression in slopes from 841.64 to 1677.70, reflecting the increasing incidence and rapid growth of gun violence across HOLC grades. The slope represents the mean annual increase in gun violence rates per 100,000 residents, calculated using least squares regression to provide a comparative measure of grade-specific trends. These findings indicate that neighborhoods graded C and D not only experience higher baseline rates of gun violence but also exhibit steeper upward trends over time. A statistical test for the equality of regression coefficients (Paternoster et al., 1998) confirmed significant differences in slopes across grades, emphasizing the need for targeted, localized interventions to address these disparities effectively.

**Table 1:** Gun violence incidents by HOLC grade in Milwaukee (2005–2021). This table presents the estimated population sizes, total gun violence incidents, and the mean annual increase in gun violence rates (slope) for each HOLC grade. The slope represents the average annual increase in gun violence rates per 100,000 residents, assuming linearity, calculated using least squares regression

HOLC grades	Estimated population	Gun violence incidents	Slope of gun violence trend
A	11,734	840	841.6363
B	55,768	7436	1260.37
C	190,827	37,463	1523.855
D	119,373	22,687	1677.697

**Fig. 3:**
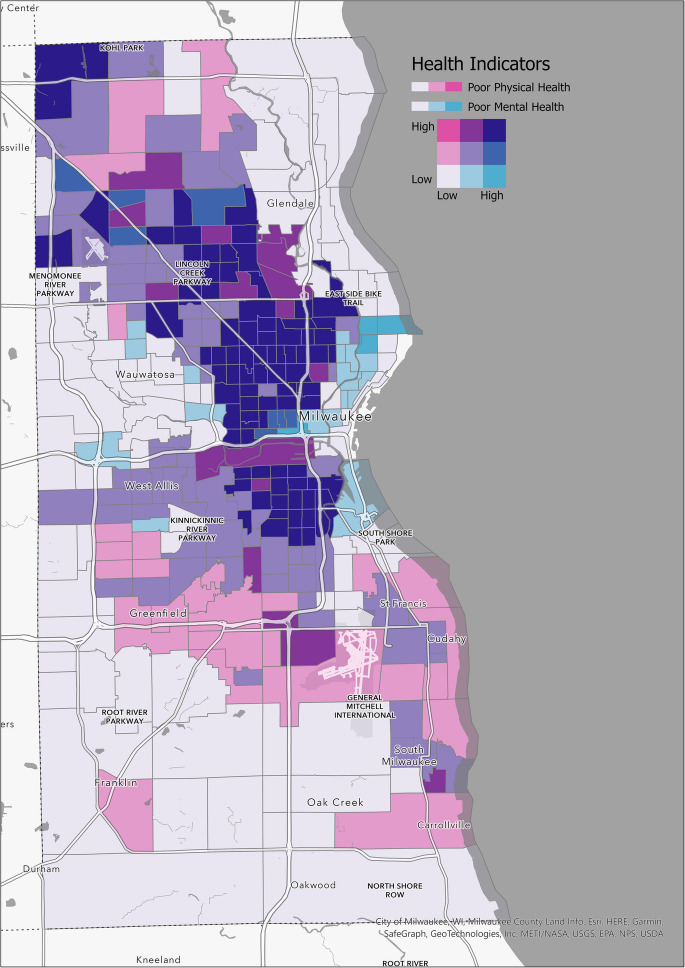
A bivariate map that visualizes the geographic areas where both poor mental and physical health outcomes overlap in Milwaukee (2014–2018). This map highlights regions where these health issues are concentrated, emphasizing the compounded impact on community well-being

Figure 3 presents a bivariate map showing the spatial distribution of poor physical and mental health outcomes in Milwaukee (2014–2018) alongside neighborhoods stratified by historical HOLC redlining grades. Neighborhoods classified as grades C and D are represented by the darkest regions, indicating the highest prevalence of these health outcomes. These areas also exhibit the highest gun violence rates (GVRs) and the steepest upward trends in gun violence. The geographic clustering of gun violence and adverse health outcomes underscores the compounded effects of historical redlining policies and socioeconomic disadvantages in these neighborhoods.

**Fig. 4:**
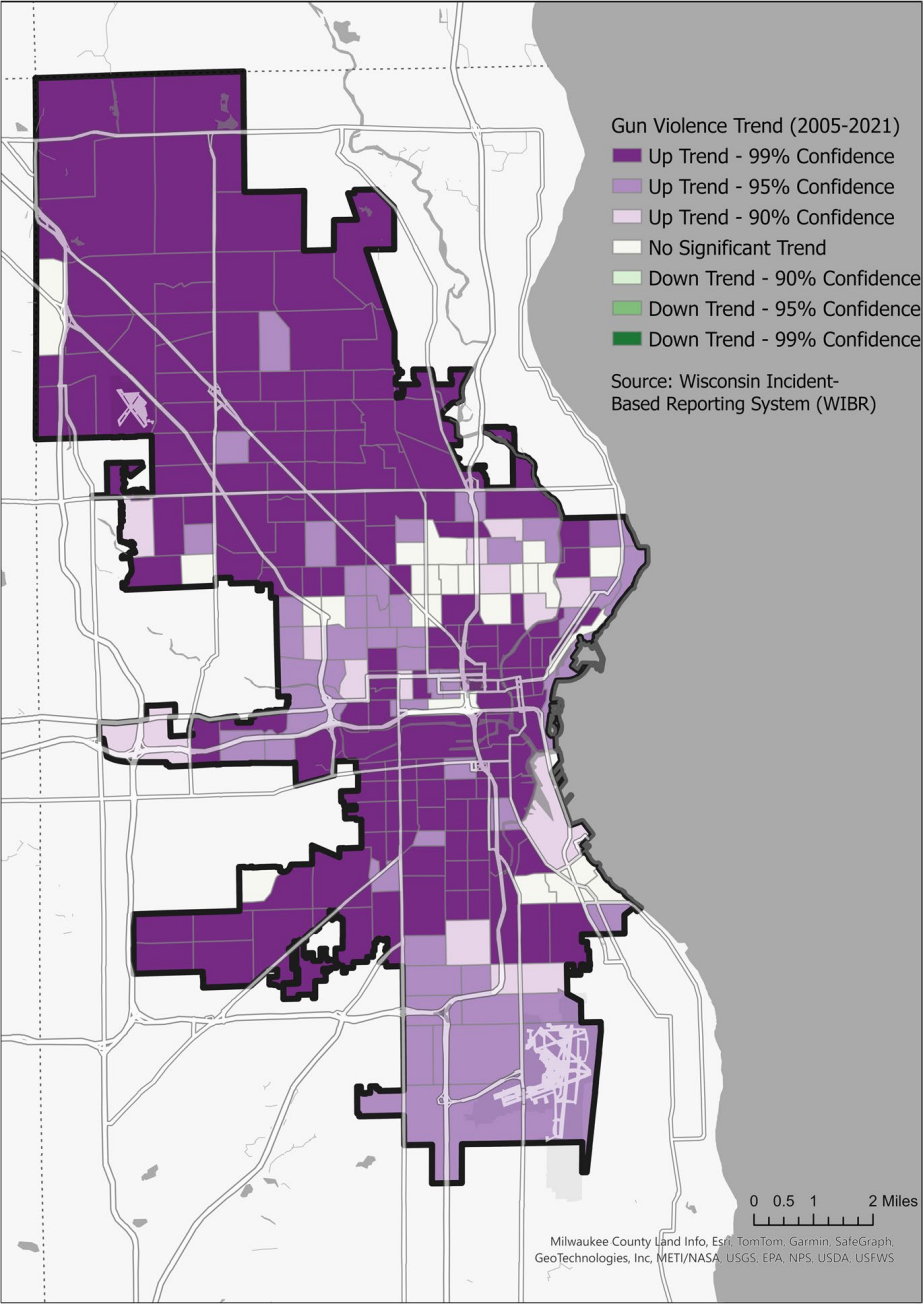
Spatial trends in gun violence across Milwaukee (2005–2021) Census tracts are classified using the Mann–Kendall trend test to detect statistically significant changes in gun violence over time. Purple shades indicateincreasing trends, green shades represent decreasing trends, and white areas show no significant change. This map reveals persistent spatial inequalities in violence trajectories across the city

Gun violence incidents aggregated at the census tract level showed a statistically significant upward trend (*p* < 0.01) across most neighborhoods (Fig. 4). The Mann–Kendall (MK) test [28] indicated a widespread increase in gun violence, reflecting a notable escalation in incident frequency over time.

The analysis revealed seven distinct spatiotemporal patterns of gun violence in Milwaukee: consecutive hot spots, new hot spots, sporadic hot spots, oscillating hot spots, persistent cold spots, diminishing cold spots, and historical cold spots. Each pattern corresponds to specific socioeconomic and demographic conditions, highlighting areas that may require tailored interventions, resource allocation, and targeted crime-prevention strategies.

As shown in Table 2, the hot spot zones with consistently high gun violence rates highlight significant socioeconomic challenges. These areas have a median household income of $35,666.28, with 31% of residents living in poverty. Health issues are prevalent, with 17.28% of the population reporting poor physical health and 19.28% experiencing mental health problems. The population is diverse, with a significant African American presence.

**Table 2:** Socioeconomic and demographic characteristics of spatiotemporal patterns of gun violence incidents in the city of Milwaukee

Pattern	White	Black	Median household income	Poverty	Poor physical health	Poor mental health	Hispanic	Employment	Educational attainment
Consecutive hot spot	16.02%	69.51%	$35,666.28	31.35%	17.28%	19.28%	11.94%	38.46%	8.64%
Historical cold spot	80.75%	6.91%	$51,253.38	18.55%	11.23%	15.41%	16.58%	56.84%	31.08%
New hot spot	54.48%	31.54%	$37,197.63	36.61%	14.74%	19.57%	19.82%	39.32%	13.19%
No pattern detected	69.35%	12.48%	$56,361.12	16.65%	11.21%	15.21%	25.95%	54.16%	27.14%
Oscillating hot spot	33.43%	44.75%	$38,475.93	30.20%	16.11%	18.16%	25.70%	43.94%	9.68%
Persistent cold spot	83.84%	4.61%	$62,871.97	14.10%	11.20%	14.50%	14.31%	54.53%	25.20%
Sporadic hot spot	10.08%	77.50%	$30,178.30	33.58%	17.74%	19.99%	8.43%	35.66%	5.97%
**City of Milwaukee**	**43.05%**	**41.31%**	**$43,711.43**	**26.42%**	**14.84%**	**17.65%**	**17.42%**	**44.86%**	**14.95%**

Regarding employment and education, 38.46% of residents are full-time, and 8.64% have attained higher education. Recent new hot spots, with increased gun violence, exhibit a median income of $37,197.63 and a high poverty rate of 36.61%, within a racially diverse area where Whites, African Americans, and Hispanics account for 54.48%, 31.54%, and 19.82% respectively. Oscillating hot spots, with fluctuating gun violence, show a median income slightly above Milwaukee’s average. In contrast, sporadic hot spots, characterized by irregular gun violence surges and a predominantly African American population, have the lowest median income and a poverty rate of 33.58%.

Contrastingly, historical cold spot regions, characterized by fewer gun violence incidents, exhibit a more prosperous socioeconomic profile, with a median income of $51,253.38 and an 18.55% poverty rate. These areas are predominantly White (80.75%), with healthier employment (56.84%) and higher education attainment (31.08%). Persistent cold spots represent the city’s affluent enclaves, boasting the highest median income at $62,871.97 and a 14.10% poverty rate.

**Fig. 5:**
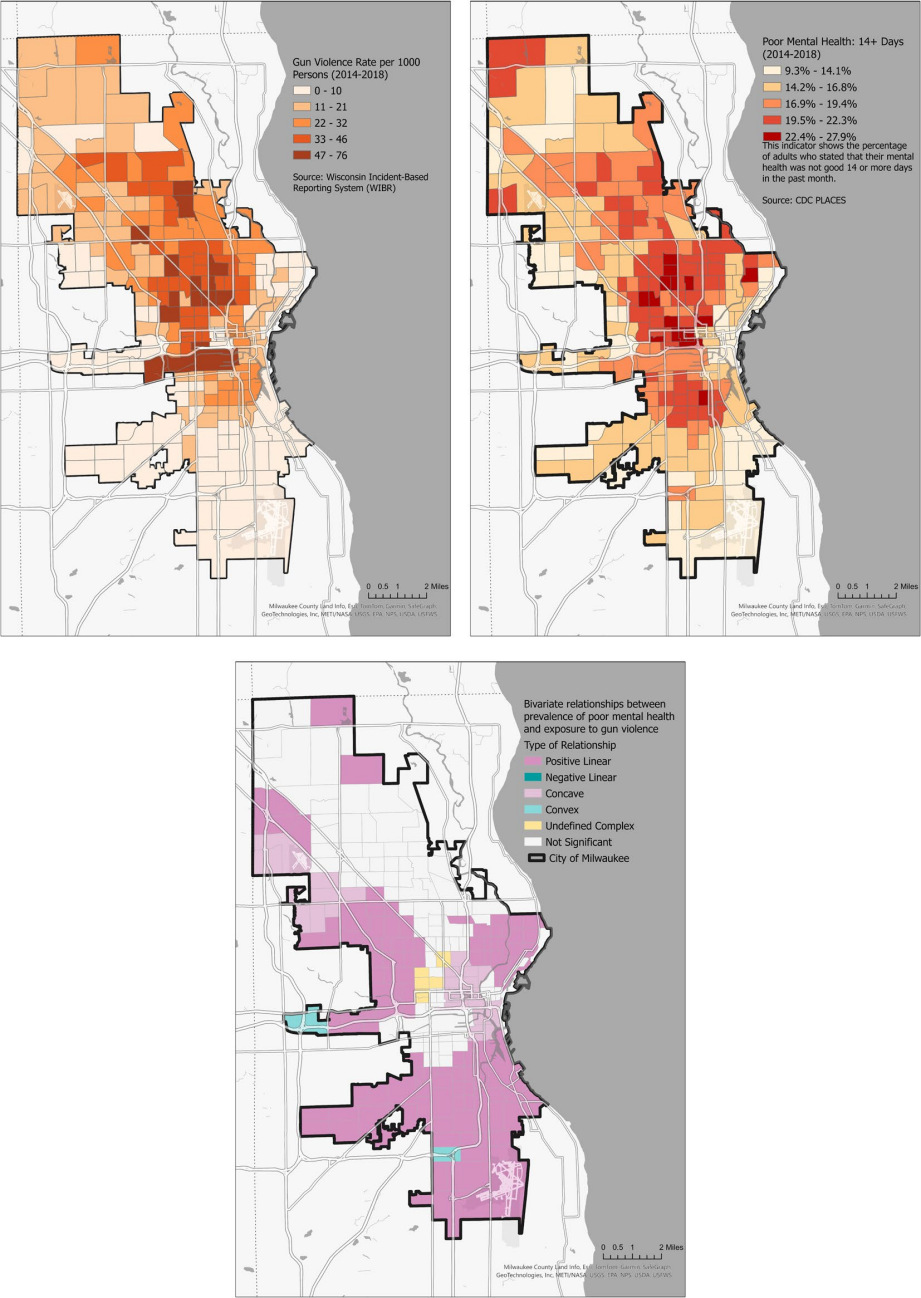
The spatial relationship between gun violence and the prevalence of poor mental health in Milwaukee (2014–2018). **a** Gun violence hotspots. **b** Areas with high prevalence of poor mental health. **c** The local correlation, revealing mostly positive linear relationships with more complex interactions in central areas

An examination of gun violence (rate per 1000 persons) alongside poor mental health (percentage of adults reporting ≥ 14 days of poor mental health in the past month) revealed distinct spatial correlations (Fig. 5). Overall, the findings indicate that neighborhood-level mental health challenges and gun violence rates are related, though the form and strength of this relationship vary across Milwaukee.

The analysis identified five relationship types between gun violence and mental health indicators: not significant, positive linear, concave, convex, and undefined complex. A “Not Significant” relationship indicates no statistically valid association, while a “Positive Linear” relationship shows that increased mental health challenges correspond with higher gun violence incidents. The “Concave” and “Convex” relationships reflect downward and upward curving connections, respectively, as mental health challenges rise. The “Undefined Complex” relationship reveals a significant correlation but does not fit the other patterns.

Most areas exhibited a positive linear or concave relationship between mental health challenges and gun-related incidents, indicating that as mental health challenges increase, gun violence also rises. However, central city neighborhoods, with the highest prevalence of poor mental health and gun violence, showed a more complex relationship that warrants further investigation. These areas, with a median income of $25,204.33, a poverty rate of 41.83%, and 61.61% African American residents, reported 21.70% poor mental health and only 5.85% with a bachelor’s degree or higher.

Areas with a positive linear trend had a higher median household income ($50,065.16) and lower poverty rate (21.07%), with a demographic composition of 58.47% Whites and 25.74% African Americans. Poor mental health was reported by 16.47%, the full-time employment rate was 49.49%, and 18.36% held a bachelor’s degree or higher. In contrast, areas with a concave relationship had a median household income of $42,999.10 and a poverty rate of 32%. These areas were evenly split between Whites (43.24%) and African Americans (45.12%). Poor mental health affected 18.07%, with 43.16% employed full-time and 18.42% holding a bachelor’s degree or higher.

The interplay of gun violence and mental health in Milwaukee is shaped by unique socioeconomic factors, necessitating targeted interventions and policies.

## Discussion

The relationship between gun violence and mental health in Milwaukee highlights the complexity of urban public health challenges, especially in racially segregated and underserved neighborhoods. This study employed entropy analysis to better understand this relationship by quantifying its uncertainty and variability. Unlike traditional linear models, entropy analysis captures patterns that are not easily identified, providing insights into the strength and nature of interdependencies between gun violence and mental health across different areas.

Our findings underscore the significant public health implications of systemic disinvestment, racial segregation, and socioeconomic inequalities in communities predominantly inhabited by racialized minorities. These areas experience higher rates of gun violence and adverse mental health outcomes, which reflect the lingering effects of structural racism. By identifying regions where these issues are most strongly interconnected, entropy analysis contributes to a broader understanding of these public health challenges.

Historically redlined neighborhoods, classified as grades C and D in the Home Owners’ Loan Corporation maps, predominantly African American and Hispanic, continue to face disproportionately high rates of gun violence and mental health disparities. These findings align with existing research linking systemic discrimination in urban planning to long-term socioeconomic disadvantages and public health crises [31], [5], [6]. The analysis highlights how these issues remain unpredictable in such neighborhoods, driven by interconnected social, economic, and historical factors. By using entropy analysis, we revealed the complexity of these relationships, enabling a deeper understanding of how they vary across neighborhoods. Moreover, the entropy analysis underscores the variability in the relationship between gun violence and mental health, revealing especially complex patterns in neighborhoods confronting poverty and limited resources. These insights highlight the need for localized, tailored interventions, particularly in areas where non-linear dynamics and disparities magnify the impact of violence on mental health.

The results align with prior studies showing the lasting impact of redlining on segregation and vulnerability. Chronic exposure to violence and limited access to mental health resources exacerbate mental health burdens, especially in high-violence areas, highlighting the need for targeted interventions [32], [33]. Interventions should be place-sensitive and culturally appropriate to address local challenges effectively and equitably.

This study calls for a shift in public health policy toward community-specific strategies that address the compounded effects of systemic inequities and violence. Expanding mental health services, reducing stigma, and fostering community cohesion are essential steps toward mitigating these challenges. Moreover, policymakers must prioritize data-driven solutions that target the physical and mental health impacts of violence, moving beyond one-size-fits-all approaches [11].

The spatial patterns of gun violence and mental health disparities emphasize the ongoing influence of historical and structural factors on contemporary public health outcomes. The neighborhoods with the highest levels of gun violence also report the poorest mental health, highlighting the interconnected nature of spatial inequality, racialized poverty, and adverse health outcomes. These findings underscore the importance of addressing geospatial and social determinants of health to promote equity [34].

Additionally, this study aligns with previous research on the psychological impacts of community violence exposure, as highlighted by Semenza et al. [35], which demonstrates the significant mental health burdens in vulnerable populations. Building on these findings, our entropy analysis quantified the unpredictability of these challenges, reinforcing the importance of tailored, localized interventions to address these disparities.

The temporal and spatial analyses of gun violence in Milwaukee further highlight the need for collaborative efforts among local governments, community groups, healthcare providers, and residents. Partnerships are essential to design and evaluate effective interventions, build community trust, and foster long-term improvements. A multidisciplinary approach is critical to addressing the root causes of violence and mental health disparities in vulnerable neighborhoods.
